# Mechanism and spectrum of inhibition of a 4′-cyano modified nucleotide analog against diverse RNA polymerases of prototypic respiratory RNA viruses

**DOI:** 10.1016/j.jbc.2024.107514

**Published:** 2024-06-28

**Authors:** Calvin J. Gordon, Simon M. Walker, Egor P. Tchesnokov, Dana Kocincova, Jared Pitts, Dustin S. Siegel, Jason K. Perry, Joy Y. Feng, John P. Bilello, Matthias Götte

**Affiliations:** 1 Department of Medical Microbiology and Immunology, University of Alberta, Edmonton, Alberta, Canada; 2 Gilead Sciences, Inc, Foster City, California, USA

**Keywords:** pandemic preparedness, prototypic pathogens, respiratory viruses, RNA virus, RNA-dependent RNA polymerase, antiviral, nucleotide analog, remdesivir, SARS-CoV-2, COVID-19, coronavirus, rhinovirus, enterovirus, respiratory syncytial virus, influenza virus

## Abstract

The development of safe and effective broad-spectrum antivirals that target the replication machinery of respiratory viruses is of high priority in pandemic preparedness programs. Here, we studied the mechanism of action of a newly discovered nucleotide analog against diverse RNA-dependent RNA polymerases (RdRps) of prototypic respiratory viruses. GS-646939 is the active 5′-triphosphate metabolite of a 4ʹ-cyano modified *C*-adenosine analog phosphoramidate prodrug GS-7682. Enzyme kinetics show that the RdRps of human rhinovirus type 16 (HRV-16) and enterovirus 71 incorporate GS-646939 with unprecedented selectivity; GS-646939 is incorporated 20-50-fold more efficiently than its natural ATP counterpart. The RdRp complex of respiratory syncytial virus and human metapneumovirus incorporate GS-646939 and ATP with similar efficiency. In contrast, influenza B RdRp shows a clear preference for ATP and human mitochondrial RNA polymerase does not show significant incorporation of GS-646939. Once incorporated into the nascent RNA strand, GS-646939 acts as a chain terminator although higher NTP concentrations can partially overcome inhibition for some polymerases. Modeling and biochemical data suggest that the 4ʹ-modification inhibits RdRp translocation. Comparative studies with GS-443902, the active triphosphate form of the 1′-cyano modified prodrugs remdesivir and obeldesivir, reveal not only different mechanisms of inhibition, but also differences in the spectrum of inhibition of viral polymerases. In conclusion, 1ʹ-cyano and 4ʹ-cyano modifications of nucleotide analogs provide complementary strategies to target the polymerase of several families of respiratory RNA viruses.

Respiratory RNA viruses represent a substantial public health burden worldwide. Facile transmission from person to person can cause outbreaks, epidemics, or pandemics. Severe acute respiratory syndrome coronavirus 2 (SARS-CoV-2), the causative agent of coronavirus disease 2019 (COVID-19) (1, 2), is a most recent example. Other prominent examples include members of the *Coronaviridae* such as SARS-CoV and Middle East respiratory syndrome coronavirus (MERS-CoV); the *Picornaviridae*, *e.g.* human rhinovirus (HRV); the *Pneumoviridae*, *e.g.* respiratory syncytial virus (RSV); the *Paramyxoviridae*, *e.g.* human parainfluenza viruses (HPIVs); and influenza viruses of the *Orthomyxoviridae* family. Infection with these pathogens is associated with diverse disease outcomes, from asymptomatic or mild sequelae to viral bronchiolitis and pneumonia. High rates of hospitalizations and mortality from viral respiratory infections are of particular concern in children, older adults, and those with chronic airway inflammatory diseases, such as asthma (3, 4, 5, 6, 7, 8).

The development of effective medical countermeasures is challenging due to the diverse nature of the aforementioned viruses that cover positive-sense RNA viruses (coronaviruses and picornaviruses), nonsegmented negative-sense RNA viruses (pneumoviruses and paramyxoviruses) and segmented negative-sense RNA viruses (orthomyxoviruses). A common target for pharmaceutical intervention strategies is the viral RNA-dependent RNA polymerase (RdRp), which is required for viral genome replication. Although the structural details of these enzymes differ across virus families and to a lesser degree from virus species to species, the active site is relatively conserved to accommodate NTP substrates. The development of antiviral nucleoside or nucleotide analogs is therefore a logical strategy to identify therapeutics with potential for broad-spectrum antiviral activity. Once incorporated into the growing RNA chain, the nucleotide analog can cause inhibition of RNA synthesis. The detailed mechanism of action depends on both the nature of the inhibitor and the nature of the polymerase.

Remdesivir (RDV) is a 1ʹ-cyano modified *C*-adenosine monophosphate prodrug (Fig. 1*A*) derived from the parent nucleoside GS-441524 (Fig. 1*B*) (9). RDV was the first antiviral drug to receive approval from the US Food and Drug Administration for the treatment of COVID-19 (10). *In vitro*, RDV and GS-441524 exhibit broad-spectrum antiviral activity against respiratory viruses, including coronaviruses (SARS-CoV-2, SARS-CoV, and MERS-CoV) (11, 12, 13, 14, 15), picornaviruses (HRV-16, enterovirus D68 [EV-D68]) (16, 17), pneumoviruses (RSV, human metapneumovirus [HMPV]) (18, 19), and HPIV (19, 20). Antiviral activity is not observed against several segmented negative-sense RNA viruses, including Lassa virus (LASV), Crimean-Congo hemorrhagic fever virus, and influenza viruses (17, 19, 20). The 5ʹ ester prodrug of GS-441524, obeldesivir (Fig. 1*C*), is metabolized presystemically to GS-441524 and subsequently to GS-443902, and therefore shares the same antiviral profile as RDV and its parent nucleoside (21). Obeldesivir was recently evaluated in phase 3 clinical trials for COVID-19 and was efficacious against filoviruses in a post-exposure prophylaxis nonhuman primate model (22).

**Figure 1:**
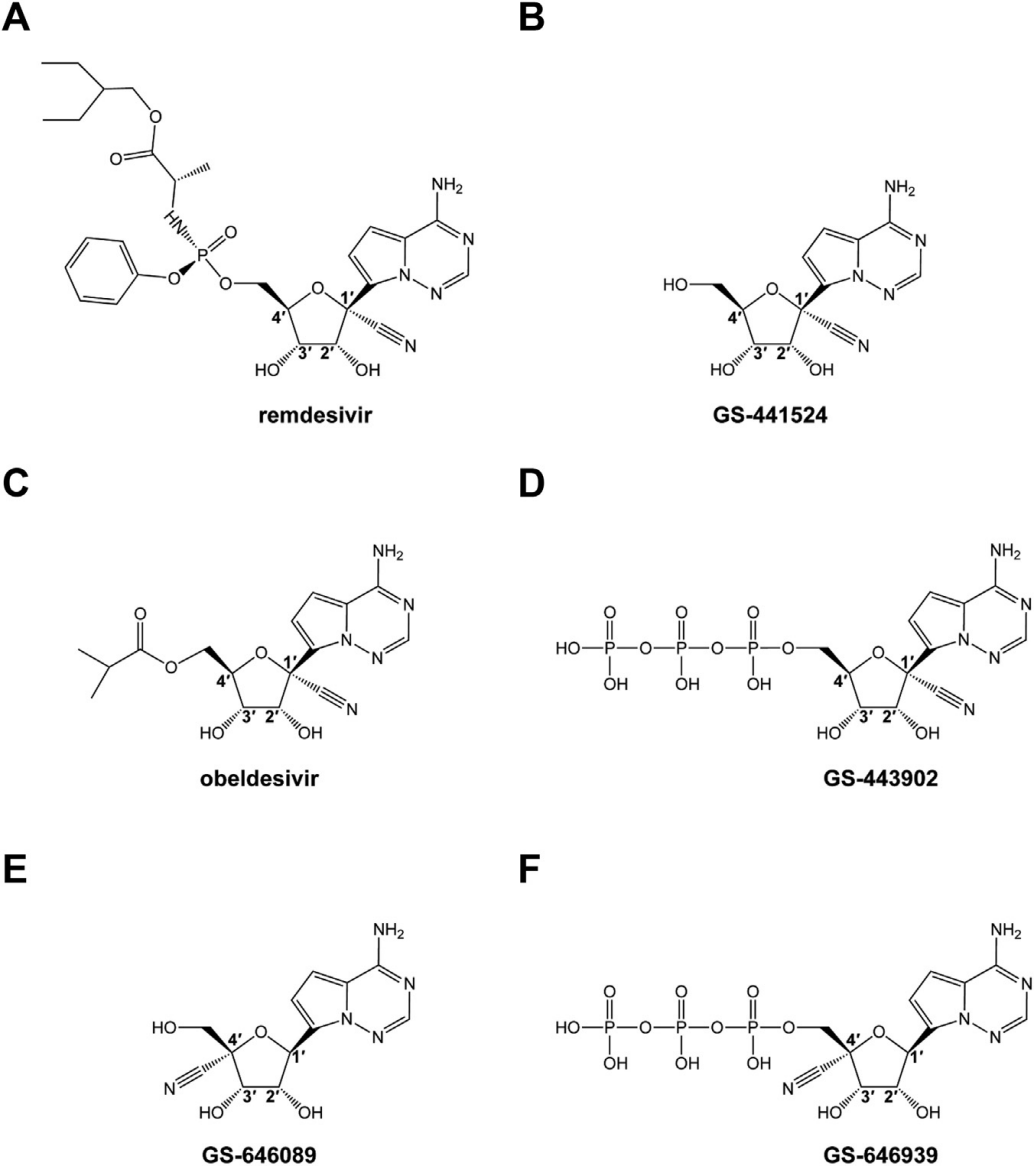
**Chemical structures ofcompounds discussed in this study.** Remdesivir (*A*), GS-441524 (*B*), obeldesivir (*C*), GS-443902 (*D*), GS-646089 (*E*), and GS-646939 (*F*)**.**

Key biochemical attributes of RDV and GS-441524 that enable potent inhibition of the SARS-CoV-2 RdRp complex have been identified. Their active 5′-triphosphate metabolite, herein referred to as GS-443902 (Fig. 1*D*), outcompetes its natural counterpart ATP 2- to 3-fold (23, 24, 25). Incorporation into the growing RNA chain at position “i” results in delayed chain-termination at position “i + 3”. A steric clash between the 1ʹ-cyano group of GS-443902 and the conserved Ser-861 in the RdRp causes inhibition of primer extension (26, 27). Higher NTP concentrations can overcome delayed chain termination at position “i + 3”, potentially yielding complete copies of the viral genome with embedded GS-443902 residues (26, 27, 28, 29, 30, 31). When used as templates, incorporation of the complementary UTP is likewise inhibited. This template-dependent inhibition is more effective, but higher NTP concentrations can overcome this obstacle as well (27).

Here, we compare the 1ʹ-cyano modified *C*-adenosine with a newly discovered 4ʹ-cyano modified *C*-adenosine. Derived from the nucleoside GS-646089 (Fig. 1*E*), the monophosphate prodrug GS-7682 is associated with a broad-spectrum of antiviral activities (32). Viruses that belong to the *Picornaviridae* and *Pneumoviridae* families were most sensitive to GS-7682 treatment in cell culture. The EC_50_ of GS-7682 was less than 100 nM against several picornaviruses, including HRV-16 (32). Further, GS-7682 was potent against HMPV and RSV with EC_50_ values of 210 ± 50 and 3 to 46 nM (various assays), respectively. However, GS-7682 demonstrated limited antiviral activity against corona- and orthomyxoviruses (32). In this study, we compared the biochemical properties of the active 5′-trisphosphate form of GS-7682, referred to as GS-646939 (Fig. 1*F*), with GS-443902 against an array of RdRp enzymes representing multiple families of respiratory viruses. Highly efficient rates of GS-646939 incorporation are seen with picornaviruses HRV-16 and EV-71 RdRp and, to a lesser extent, with pneumoviruses RSV and HMPV RdRp. In contrast to GS-443902, inhibition with GS-646939 is based on immediate chain-termination at position “i”. Overall, the biochemical evaluation of the two nucleotide analogs against their targets provides mechanistic detail for the observed antiviral effects.

## Results

### Experimental strategy

The main objective of this study was to elucidate the mechanism of action of GS-646939 using purified RdRp enzymes from prototypic respiratory RNA viruses of *Coronaviridae*, *Picornaviridae*, *Pneumoviridae*, *Paramyxoviridae* and *Orthomyxoviridae* (Table 1). The prototypic pathogen approach is based on research efforts with selected viruses that represent specific families (33). To strengthen this approach, we expressed the RdRp from two prototypic species within each of the five families mentioned above. SARS-CoV-2 and MERS-CoV were selected to represent coronaviruses, HRV-16 and EV-71 for picornaviruses, RSV and HMPV for pneumoviruses, and HPIV-3 and PIV-5 for paramyxoviruses. Orthomyxoviruses were represented by influenza B virus. The RdRp from LASV, a segmented negative-sense RNA virus (like influenza), which belongs to the *Arenaviridae* family, was also evaluated. Human mitochondrial RNA polymerase (h-mtRNAP) was used to assess potential off-target effects. The comprehensive approach was designed to identify common biochemical properties that are associated with broad-spectrum antiviral activity. Active RdRp enzymes used in this study exist either as monomers, dimers, or multimeric complexes (Table 1). The coronaviruses SARS-CoV-2 and MERS-CoV form complexes with nonstructural protein 12 (Nsp12), which contains the RdRp active site, and essential cofactors Nsp7 and Nsp8 (25, 34, 35). Picornavirus RdRps from HRV-16 and EV-71, also referred to as 3D^pol^, are monomeric and do not require additional cofactors (36, 37). The replication complex of the pneumoviruses RSV and HMPV, as well as paramyxoviruses HPIV-3 and PIV-5, consists of the large (L) protein, containing the RdRp active site, and a phosphoprotein (P) (38, 39). PIV-5, or canine parainfluenza virus, was included because the structure of the RdRp complex is available (40) (Table 1). While this manuscript was under review, the structure of the HPIV-3 polymerase complex was also reported (41). Influenza viruses assemble a heterotrimeric complex composed of PA (cap-snatching endonuclease subunit), PB1 (RdRp subunit), and PB2 (cap-binding subunit) (42). The LASV L protein is a dynamic monomer functionally similar to the trimeric influenza replication complex, possessing an N-terminus endonuclease, RdRp core, and C-terminus cap-binding domain (43). Finally, h-mtRNAP is a single subunit enzyme responsible for the transcription of the mitochondrial genome (44). In this study, we combined enzymatic assays with structural modeling to provide insight into the requirements for nucleotide incorporation and inhibition of RNA synthesis, respectively, with GS-443902 serving as a benchmark.

**Table 1:** Expression of RNA polymerases

Sense	Family	Virus	Expression system	Construct	Composition	M.W. (kDa)^a^	PDB
Positive ssRNA	*Coronaviridae*	SARS-CoV-2	BEVS^b^	nsp5-↓^c^ nsp7-↓ 8xHis-nsp8-↓**nsp12**^d^↓	Tetramer	165	7UO4^e^
		MERS-CoV		nsp5-↓ nsp7-↓ 8xHis-nsp8-↓**nsp12**-Strep	Tetramer	165	n/a
	*Picornaviridae*	HRV-16	*E. coli*	8xHis-**3D**^**pol**^	Monomer	50	4K50
		EV-71				50	3N6L
Nonsegmented negative ssRNA	*Pneumoviridae*	RSV	BEVS	TEV-↓ 8xHis-P-↓**L**-Strep	Dimer	282	6PZK
		HMPV		TEV-↓ 8xHis-P-↓ Strep-**L**		267	6U5O
	*Paramyxoviridae*	HPIV-3^f^	BEVS	^p10^P-^pPH^**L**-8xHis	Dimer	323	8KDB
		PIV-5^f^				298	6V85
Segmented negative ssRNA	*Orthomyxoviridae*	FluB	BEVS	TEV-↓ 8xHis-↓ PA-↓**PB1**-↓ PB2-Strep	Trimer	263	6QCV
	*Arenaviridae*	LASV	BEVS	Strep-8xHis-**L**	Monomer	257	7OJN
Human DNA-dependent RNA polymerase		h-mtRNAP	BEVS	Strep-8xHis-**RNAP**	Monomer	138	4BOC

TEV, tobacco etch virus protease.

a Molecular weight is an approximation and calculated according to the subunit composition of the replication complex (*e.g.* coronavirus replication complex accounts for the ratio 1:2:1 of Nsp7:Nsp8:Nsp12 as observed structurally). The protease is not included in the calculation.

b Baculovirus expression vector system.

c Down arrow represents the cleavage site acted on by that constructs respective protease (*underlined*).

d Bolded domains indicate where the RdRp motifs responsible for catalyzing RNA synthesis are contained.

e Ternary structure of the SARS-CoV-2 replication complex with GS-443902 in the active site as reported by Malone *et al.* (25).

f Cloned into pFastBac-DUAL vector employing the p10 and polyhedrin (pPH) promoter as reported by Abdella at el (40).

### Selective incorporation of GS-443902 and GS-646939 by representative RdRp enzymes

We compared nucleotide incorporation efficiency of the 1ʹ-cyano GS-443902 (Table 2, *column 4*) with the 4ʹ-cyano GS-646939 modified NTP (Table 2, *column 5*). Enzyme reactions were monitored in gel-based assays using model primer/templates (Fig. S1) (26, 45, 46, 47, 48). Steady-state kinetic parameters V_max_/*K*_m_ for ATP incorporation over V_max_/*K*_m_ for the nucleotide analog provide a measure of the efficiency of nucleotide incorporation in relation to its natural counterpart (Table S1 and S2). The numerical value is without a unit and defines the selectivity for a given nucleotide analog. Selectivity values below one indicate that the nucleotide analog is more efficiently incorporated than ATP. Previous studies revealed selectivity values of ∼0.3 for GS-443902 incorporation by RdRp complexes of coronaviruses SARS-CoV-2, SARS-CoV, and MERS-CoV (Table 2, *column 4*) (23, 26, 27, 45). HRV-16 and EV-71 RdRp show selectivity values around one for GS-443902, indicating that picornavirus enzymes incorporate this nucleotide analog and ATP with similar efficiency. RdRp complexes of pneumoviruses RSV and HMPV show higher selectivity values of 2.7 and 6.7, respectively, signifying a preference for the natural ATP nucleotide. A similar observation was made with paramyxovirus enzyme complexes of HPIV-3 (7.2) and PIV-5 (8.3). Incorporation of GS-443902 by influenza B and LASV RdRp is even more limited, with selectivity values of 68 and 20, respectively. GS-443902 incorporation by h-mtRNAP is highly inefficient, with a selectivity value of ∼500 (47).

**Table 2:** Selective incorporation of GS-443902 and GS-646939, by selected RdRp enzymes

RNA sense	Family	Virus	GS-443902	GS-646939
			Selectivity^a^ (fold)	Selectivity^b^ (fold)
Positive ssRNA	*Coronaviridae*	SARS-CoV-2	0.28^d^	1.37
		SARS-CoV	0.32^d^	N.R.^c^
		MERS-CoV	0.35^d^	1.17
	*Picornaviridae*	HRV-16	1.03	0.018
		EV-71	0.93	0.051
Nonsegmented negativessRNA	*Pneumoviridae*	RSV	2.73^e^	1.58
		HMPV	6.71	1.73
	*Paramyxoviridae*	HPIV-3	7.20	10.3
		PIV-5	8.31	5.05
Segmented Negative ssRNA	*Orthomyxoviridae*	FluB	68^d^	2288
	*Arenaviridae*	LASV	20^d^	225
Human DNA-dependent RNA polymerase		h-mtRNAP	503^e^	1538

a Selectivity of a viral RNA polymerase for a GS-443902 is calculated as the ratio of the V_max_/*K*_m_ values for ATP and GS-443902 analog, respectively.

b Selectivity of a viral RNA polymerase for a GS-646939 is calculated as the ratio of the V_max_/*K*_m_ values for ATP and GS-646939 analog, respectively.

c Not reported.

d Data from Gordon *et al.* (45).

e Data from Tchesnokov *et al.* (47).

GS-646939 shows incorporation selectivity values of approximately 1.2 to 1.4 for SARS-CoV-2 and MERS-CoV RdRp (Table 2, *column 5*). For HRV-16 and EV-71 RdRp, GS-646939 selectivity values are as low as 0.018, markedly exceeding the incorporation efficiency of the 1ʹ-cyano GS-443902 by up to ∼50-fold. RSV and HMPV RdRp show selectivity values of ∼1.5 for GS-646939, a slight improvement over GS-443902. Incorporation by HPIV-3 and PIV-5 RdRp show comparable selectivity values between 5 and 10 for both nucleotide analogs. Similar to GS-443902, GS-646939 served as a poor substrate for influenza B and LASV RdRp, generating selectivity values greater than 200. Likewise, h-mtRNAP demonstrated poor incorporation efficiency of GS-646939 with a selectivity value of ∼1500. Overall, GS-646939 and GS-443902 follow similar trends. The exception is the preference for GS-646939 by picornavirus RdRp and a predilection for GS-443902 by coronavirus RdRp.

**Figure 2:**
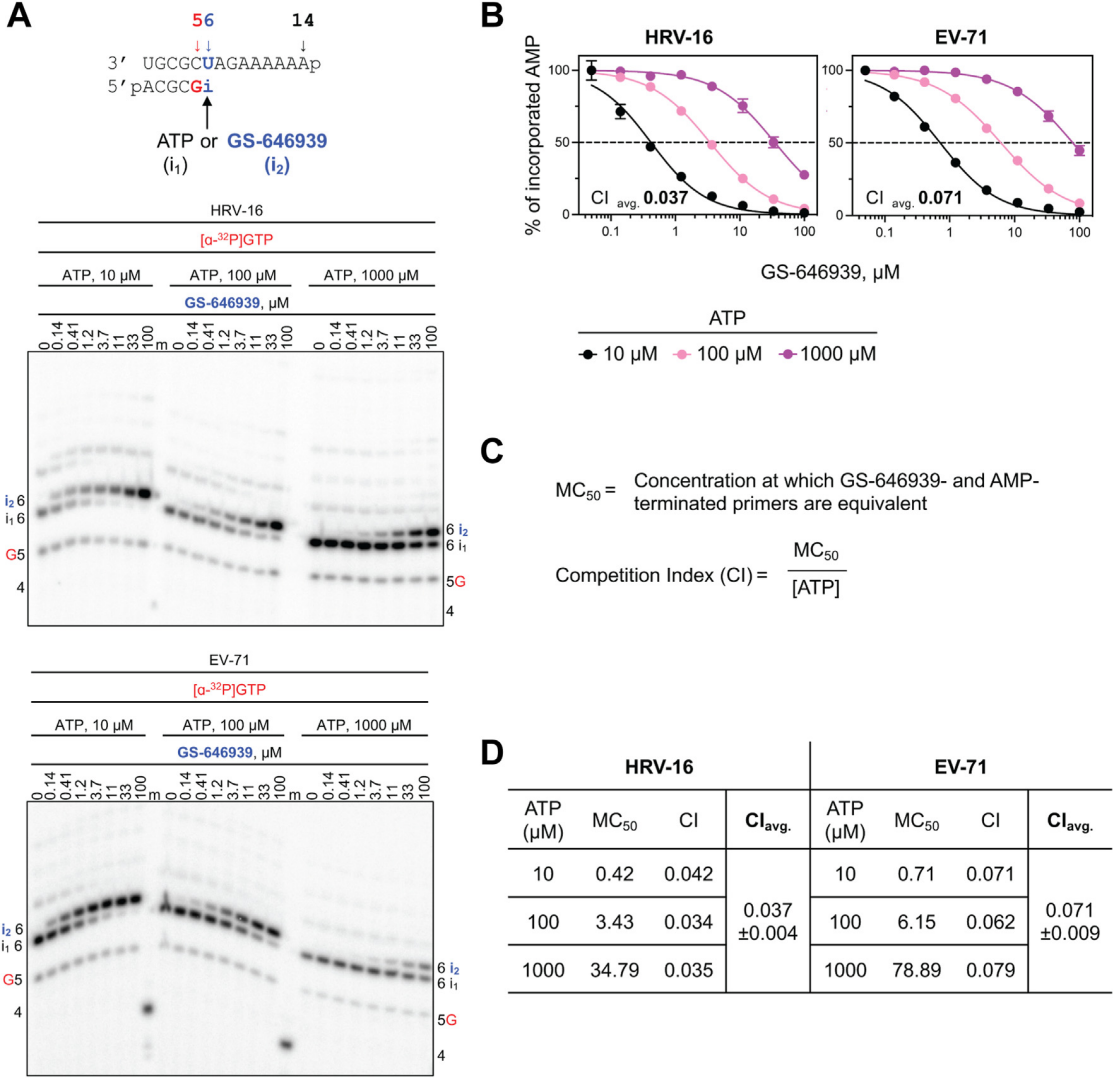
**Incorporation of GS-646939 under competitive conditions by HRV-16 and EV-71 RdRp**. *A*, RNA primer/template (*top*) supporting a single incorporation of ATP (“i_1_”) or GS-646939 (“i_2_”) at position 6. G indicates incorporation of [α-^32^P]-GTP at position 5. Migration pattern of RNA synthesis products catalyzed by HRV-16 (*middle*) and EV-71 RdRp (*bottom*). Product formation resulting in AMP- or GS-646939-terminated primers was compared across increasing GS-646939 concentrations at ATP concentrations of 10, 100, and 1000 μM. A 5′-^32^P-labeled 4-nt primer serves as a size marker. *B*, graphical representation of AMP-terminated primers (%) at increasing GS-646939 concentrations as shown in (A). Independent 8-data point experiments were performed at least three times (n = 3) and error bars represent standard error associated with the fit. *C*, the MC_50_ value is defined as the concentration at which GS-646939 matches ATP for incorporation at position 6. The competition index (CI) is the ratio of the MC_50_ value to the ATP concentration present in the reaction. *D*, CI values determined for HRV-16 and EV-71 RdRp and the CI average (CI_avg_) and standard deviation (±) across all ATP concentrations. RdRp, RNA-dependent RNA polymerase; HRV, human rhinovirus; EV-71, enterovirus 71; MC_50_, matching concentration.

The exceptional substrate utilization of GS-646939 by HRV-16 and EV-71 RdRp suggests that the nucleotide analog can outcompete its natural counterpart ATP. To address this question directly, we simultaneously added ATP and GS-646939 to the reaction and monitored incorporation of the corresponding monophosphate formed (Fig. 2). AMP (“i_1_”) and GS-646939 (“i_2_”) terminated primers could be distinguished from one another due to the difference in migration pattern (Fig. 2*A*). The concentration of GS-646939 required to match 50% ATP incorporation is defined as the matching concentration (MC_50_). As expected for a competitive inhibitor, the MC_50_ value increased with increasing concentrations of the competing ATP (Fig. 2*B*). The ratio of the MC_50_ value over the ATP concentration provides the competition index (Fig. 2*C*). The lower the value, the better the competitive advantage for incorporation of a given nucleotide. The average competition index values for GS-646939 were 0.04 and 0.07 for HRV-16 and EV-71 RdRp, respectively (Fig. 2*D*). These data suggest that, under competitive conditions, the picornavirus RdRp enzymes use GS-646939 ∼14- to 25-fold more efficiently than ATP. Furthermore, both picornavirus enzymes demonstrated a greater than 30-fold preference for GS-646939 over GS-443902 under competitive conditions (Fig. S2). Together, the competitive incorporation data corroborate the selectivity measurements.

### Structural models of incorporation

**Figure 3:**
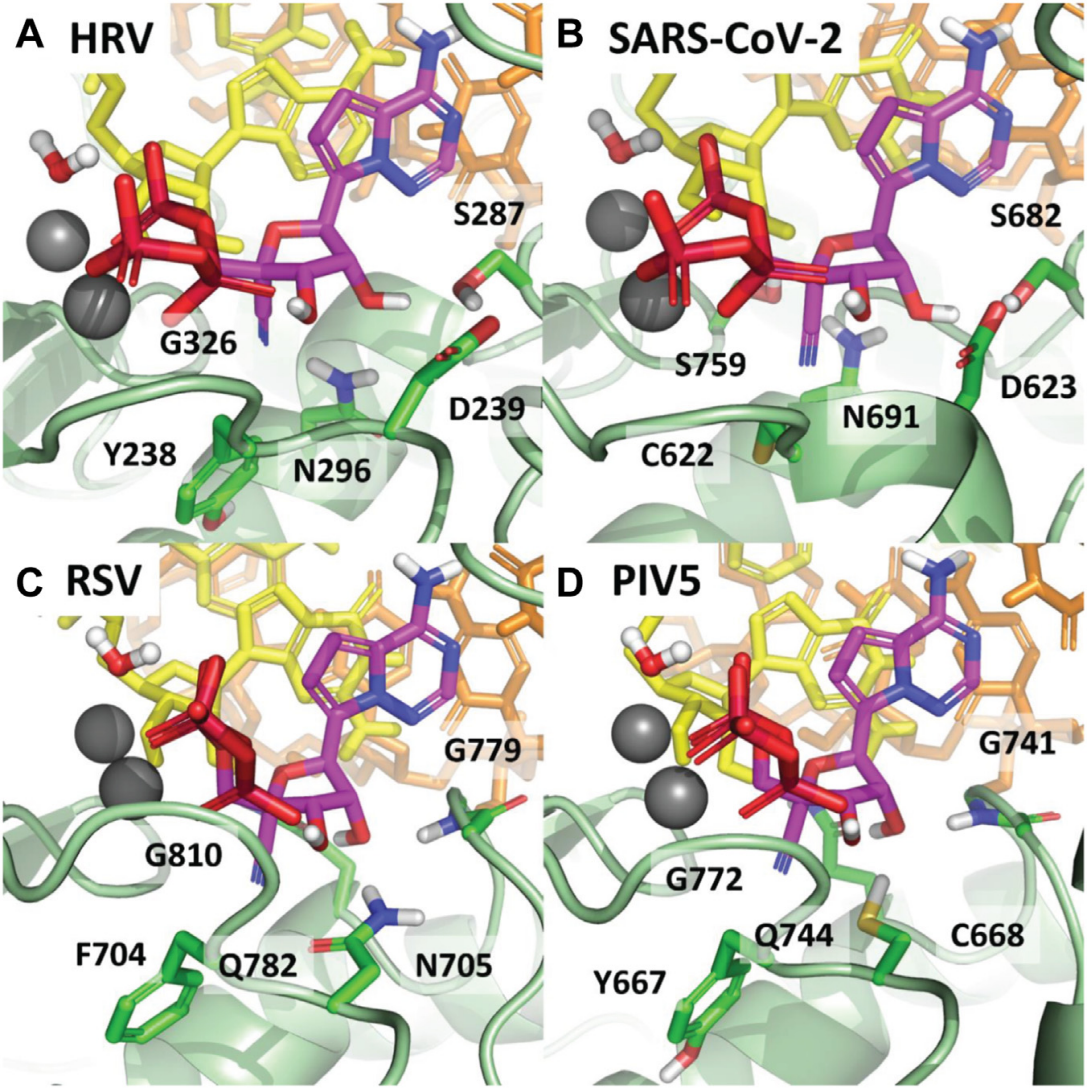
**Largely driven by the nature of the 4’ pocket and the specifics of how the 2’-OH is recognized by the polymerase.** GS-646939 is shown in magenta. HRV (*A*) and SARS-CoV-2 (*B*) have a similar overall active site structure, differing primarily at Gly-326/Ser-759 and Tyr-238/Cys-622. While somewhat different from HRV and SARS-CoV-2, RSV (*C*) and PIV5 also have a similar overall structure, differing primarily at Phe-704/Tyr-667 and Asn-705/Cys-668. In each case, the 4ʹ-cyano of GS-646939 is at least tolerated. The interaction between the 4ʹ-cyano and Asn-296 in HRV appears to be particularly ideal and is likely responsible for the inhibitor’s increased affinity compared to ATP. HRV, human rhinovirus; PIV-5, parainfluenza virus 5; RSV, respiratory syncytial virus; SARS-CoV-2, severe acute respiratory syndrome coronavirus 2.

Based on existing X-ray and cryo-EM structures (25, 36, 38, 40, 42, 43, 44), we generated models of the preincorporated states of ATP, GS-443902, and GS-646939 for the representative RdRp enzymes investigated in this study (Table 1). The active site of viral RdRps is generally characterized by a set of motifs with highly conserved residues involved in substrate binding and catalysis (49). However, subtle variations in the active site landscape seem to govern the specificity of inhibitor incorporation. For HRV-16, EV-71, SARS-CoV-2, MERS-CoV, RSV, HMPV, PIV-5, and HPIV-3, both the 1ʹ-cyano of GS-443902 and the 4ʹ-cyano of GS-646939 are tolerated to varying degrees. As previously described, the 1ʹ-cyano of GS-443902 is particularly well positioned in the coronavirus active site, occupying a uniquely polar pocket defined by Thr-687, Asn-691, and Ser-759 in SARS-CoV-2 (25), and similarly for MERS-CoV. For GS-646939, the 4′-cyano appears to have a favorable interaction with Asn-296 of HRV-16 (Fig. 3*A*). The Motif C residue Gly-326 in HRV-16 allows for maximum flexibility in the 4ʹ pocket, in contrast to the similar SARS-CoV-2 and MERS-CoV pocket, in which the corresponding residue is a serine (Fig. 3*B*). For RSV (Fig. 3*C*) and HMPV, PIV-5 (Fig. 3*D*) and HPIV-3, the 4ʹ-cyano fits nicely in the pocket, but does not appear to convey any advantage.

For LASV and FluB, both the 1ʹ-cyano of GS-443902 and 4ʹ-cyano of GS-646939 disrupt a water-mediated hydrogen bond network, which is essential for recognition of the substrate 2ʹ-OH (Fig. S3, *A* and *B*). For GS-646939, the water molecule is likely completely displaced, with no means to compensate for the loss in hydrogen bonding at 2′. Compounding the issue, both LASV and FluB have a bulky tryptophan residue forming the floor of the 4ʹ pocket. The comparable residue in the polymerases for which GS-646939 has demonstrably greater activity is typically a tyrosine, phenylalanine, or another smaller residue. This provides a degree more flexibility to accommodate the 4ʹ-cyano. In the case of h-mtRNAP, motif B is fundamentally different from viral RdRps (Fig. S3*C*). With respect to GS-443902, the 1ʹ pocket is clearly occluded by His-1125, while for GS-646939, the 4ʹ pocket is occluded by a salt bridge formed from Arg-802 and Asp-1128. In both cases, incorporation of the inhibitor should be significantly compromised.

### Inhibition of RNA primer extension reactions

**Figure 4:**
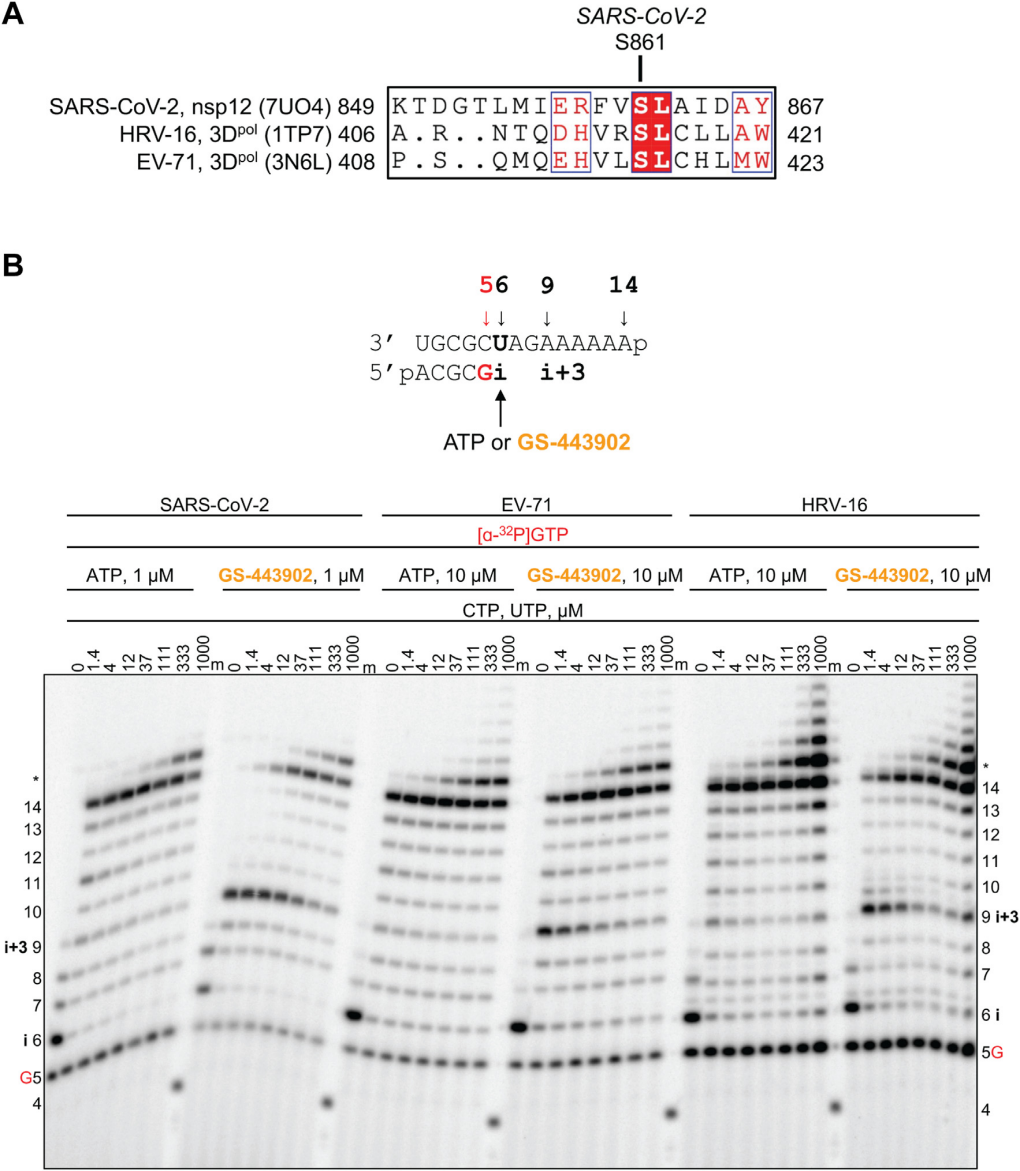
**SARS-CoV-2, EV-71, and HRV-16 RdRp-catalyzed RNA synthesis pattern of inhibition following a single incorporation of ATP or GS-443902 as a function of nucleotide concentration**. *A*, sequence alignment based on a 3D structural overlay of SARS-CoV-2 Nsp12 (PDB:7UO4), HRV-16 3D^pol^ (PDB:1TP7), and EV-71 3D^pol^ (PDB:3N6L) composed using ESPript 3.0 (82). Ser-861 (SARS-CoV-2 numbering) is conserved in HRV-16 and EV-71 RdRp. *B*, RNA primer/template supporting a single incorporation of ATP or GS-443902 at position 6 (*top*). G indicates incorporation of [α-^32^P]-GTP at position 5. Extension following the incorporation of ATP and GS-443902 at position 6 (“i”) at increasing CTP and UTP concentrations catalyzed by SARS-CoV-2, EV-71, and HRV-16 RdRp (*bottom*). Following GS-443902, an intermediate product forms at position 9 (“i + 3”), which is overcome at elevated CTP and UTP concentrations. A 5′-^32^P-labeled 4-nt primer serves as a size marker. Product formation at and above the *asterisk* indicates RNA products that are likely a result of sequence-dependent slippage events. EV-71, enterovirus 71; HRV, human rhinovirus; Nsp12, nonstructural protein 12; PDB, Protein Data Bank; RdRp, RNA-dependent RNA polymerase; SARS-CoV-2, severe acute respiratory syndrome coronavirus 2.

Incorporation of the nucleotide analog into the growing RNA chain is a prerequisite for any potential inhibitory effect. Notably, GS-443902 and GS-646939 possess a 3ʹ-hydroxyl group, classifying them as nonobligate chain terminators. The presence of a 3ʹ-hydroxyl group may allow for the nucleophilic attack on the incoming nucleotide and its subsequent incorporation. Previous biochemical and structural studies of SARS-CoV-2 have established that incorporation of GS-443902 at position “i” results in delayed chain termination at position “i + 3” (26, 27, 28). This mechanism can be attributed to a steric clash between the 1ʹ-cyano group and the hydroxyl group of the conserved Ser-861. A structural comparison between Nsp12 of SARS-CoV-2 and the 3D^pol^ of HRV-16 and EV-71 revealed that the picornaviruses share an analogous serine (Fig. 4*A*) (29). RNA synthesis following GS-443902 incorporation by SARS-CoV-2, EV-71, and HRV-16 RdRp at position “i” generated the same intermediate product at position “i + 3” (Fig. 4*B*). As reported for SARS-CoV-2 RdRp, the inhibitory effect was favored at low nucleotide concentrations and could be overcome with increasing concentrations of the nucleotide substrate at position “i + 4” (26, 27).

**Figure 5:**
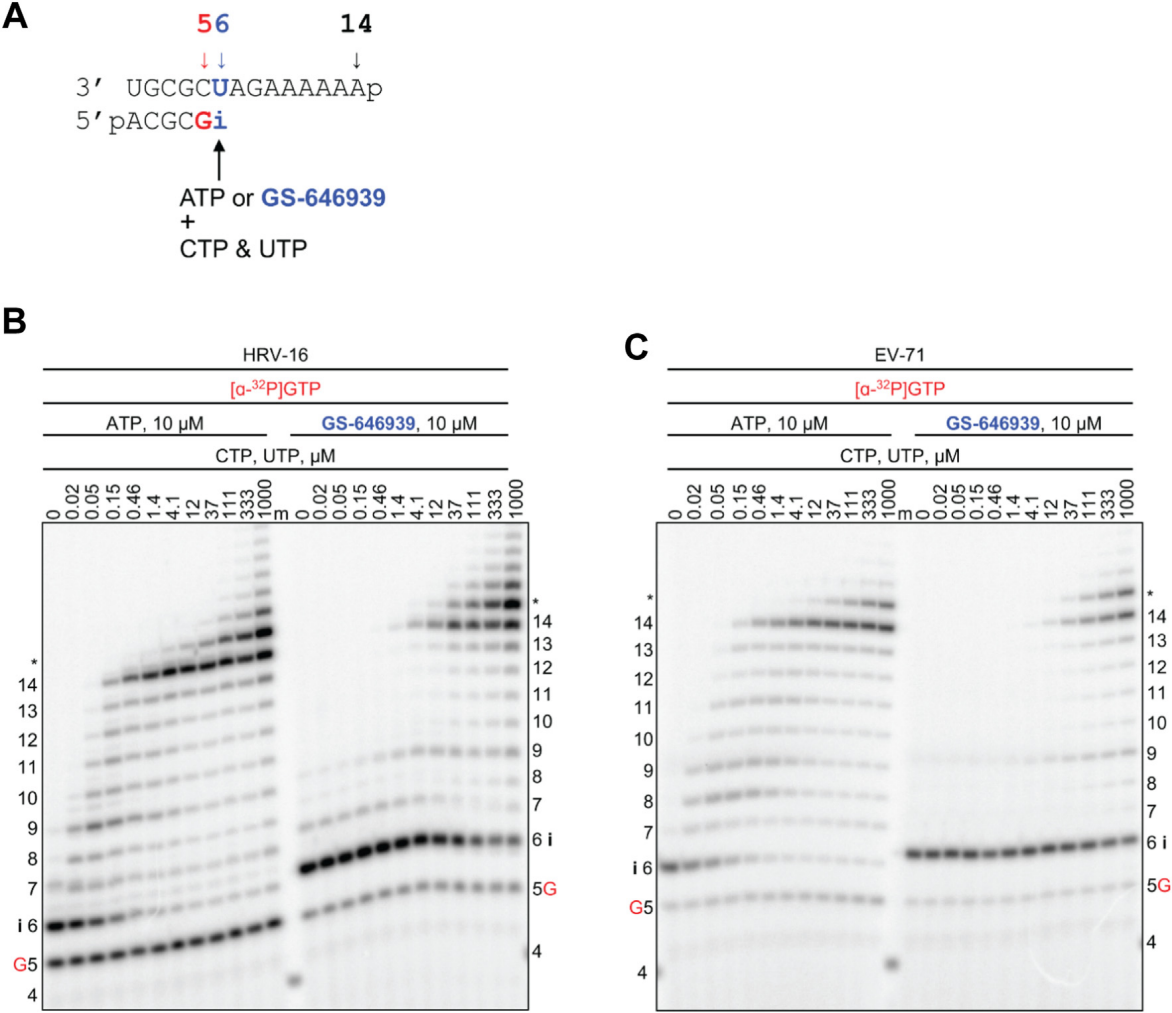
**HRV-16 or EV-71 RdRp-catalyzed RNA synthesis and pattern of inhibition following a single incorporation of ATP or GS-646939 as a function of nucleotide concentration**. *A*, RNA primer/template supporting RNA synthesis and a single incorporation of ATP or GS-646939 at position 6 (“i”). G indicates incorporation of [α-^32^P]-GTP at position 5. *B*, migration pattern of RNA products resulting from HRV-16 RdRp-catalyzed RNA extension of AMP (*left*) of GS-646939 (*right*) at increasing concentrations of CTP and UTP. A 5′-^32^P-labeled 4-nt primer serves as a size marker. Product formation at and beyond the *asterisk* indicates RNA products that are likely a result of sequence-dependent slippage events. *C*, RNA synthesis products catalyzed by EV-71 RdRp. Incomplete inhibition of RNA synthesis occurs at the site of GS-646939 incorporation (“i”), full template-length product is generated at elevated nucleotide concentrations. EV-71, enterovirus 71; HRV, human rhinovirus; RdRp, RNA-dependent RNA polymerase.

Using the same approach as above, we show that GS-646939 inhibited HRV-16 and EV-71 RdRp-mediated RNA synthesis at the site of incorporation (Fig. 5). While GS-443902 incorporation promotes delayed chain termination, GS-646939 causes immediate chain-termination. Inhibition by GS-646939 is overcome with increasing NTP concentration following the incorporated analog. A similar pattern was observed for SARS-CoV-2 and MERS-CoV RdRp (Fig. S4). GS-443902 inhibits other RdRp enzymes likewise *via* delayed chain termination. However, the patterns are more heterogeneous, with variations in the position of inhibition; the structural reasons have yet to be determined (47). In contrast, GS-646939 incorporation by RSV and HMPV RdRp resulted in immediate chain termination (Fig. S5), which was nearly absolute and could not be overcome to a discernable extent with increased NTP concentrations. Inhibition of HPIV-3 and PIV-5 RdRp with GS-646939 shows a similar immediate chain termination pattern (Fig. S6).

**Figure 6:**
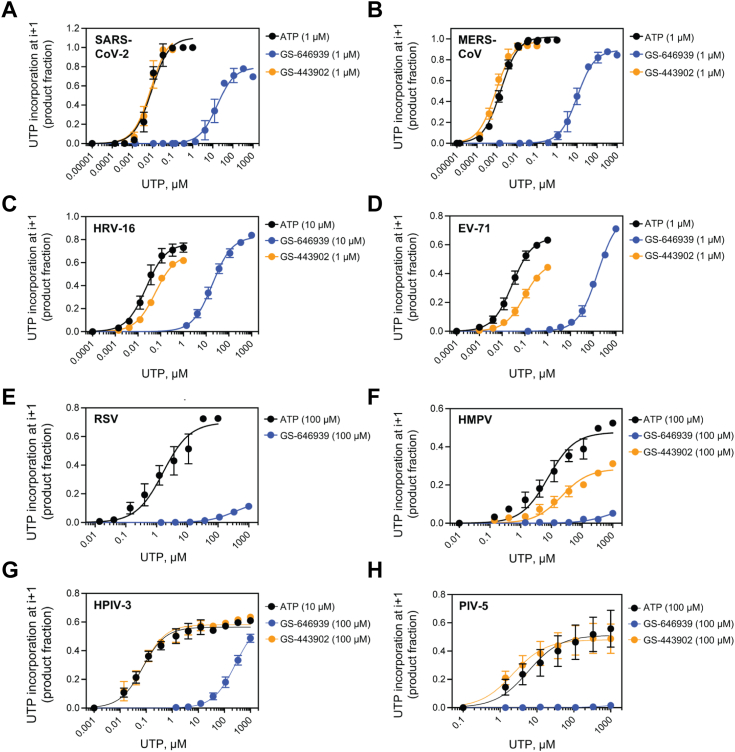
**GS-646939 inhibits subsequent nucleotide incorporation**. UTP incorporation at position “i + 1” was monitored at increasing concentrations immediately following ATP (*black*), GS-443902 (*orange*), or GS-646939 (*blue*) incorporation at position “i”. Concentrations of ATP, GS-443902, and GS-646939 supplemented to the reaction are shown in *brackets*. Viral RdRp enzymes investigated include SARS-CoV-2 (*A*), MERS-CoV (*B*), HRV-16 (*C*), EV-71 (*D*), RSV (*E*), HMPV (*F*), HPIV-3 (*G*), and PIV-5 (*H*). The product fraction was calculated as the total signal above position “i” divided by total signal in the lane. Independent 8-data point experiments were performed three times (n = 3), and error bars represent standard error associated with the fit. RdRp, RNA-dependent RNA polymerase; HRV, human rhinovirus; EV-71, enterovirus 71; RSV, respiratory syncytial virus; SARS-CoV-2, severe acute respiratory syndrome coronavirus 2; MERS-CoV, Middle East respiratory syndrome coronavirus; HMPV, human metapnuemovirus; HPIV-3, human parainfluenza virus 3; PIV-5, parainfluenza virus 5.

To quantify the inhibitory effect of GS-443902 and GS-646939 on subsequent nucleotide incorporation, we generated AMP-, GS-443902-, and GS-646939-terminated primers and measured kinetic parameters of the natural UTP substrate incorporation at position “i + 1” (Fig. 6 and Table S3). For all RdRp enzymes, compared to AMP-terminated primers, the extension of GS-646939-terminated primers required a marked increase in UTP concentration. Conversely, GS-443902-terminated primers promoted subtle inhibition of UTP incorporation for HRV-16, EV-71, RSV, and HMPV RdRp. For SARS-CoV-2, MERS-CoV, HPIV-3, and PIV-5 RdRp, equivalent or improved utilization of the GS-443902-terminated primer was observed.

### Structural rationale for chain termination

**Figure 7:**
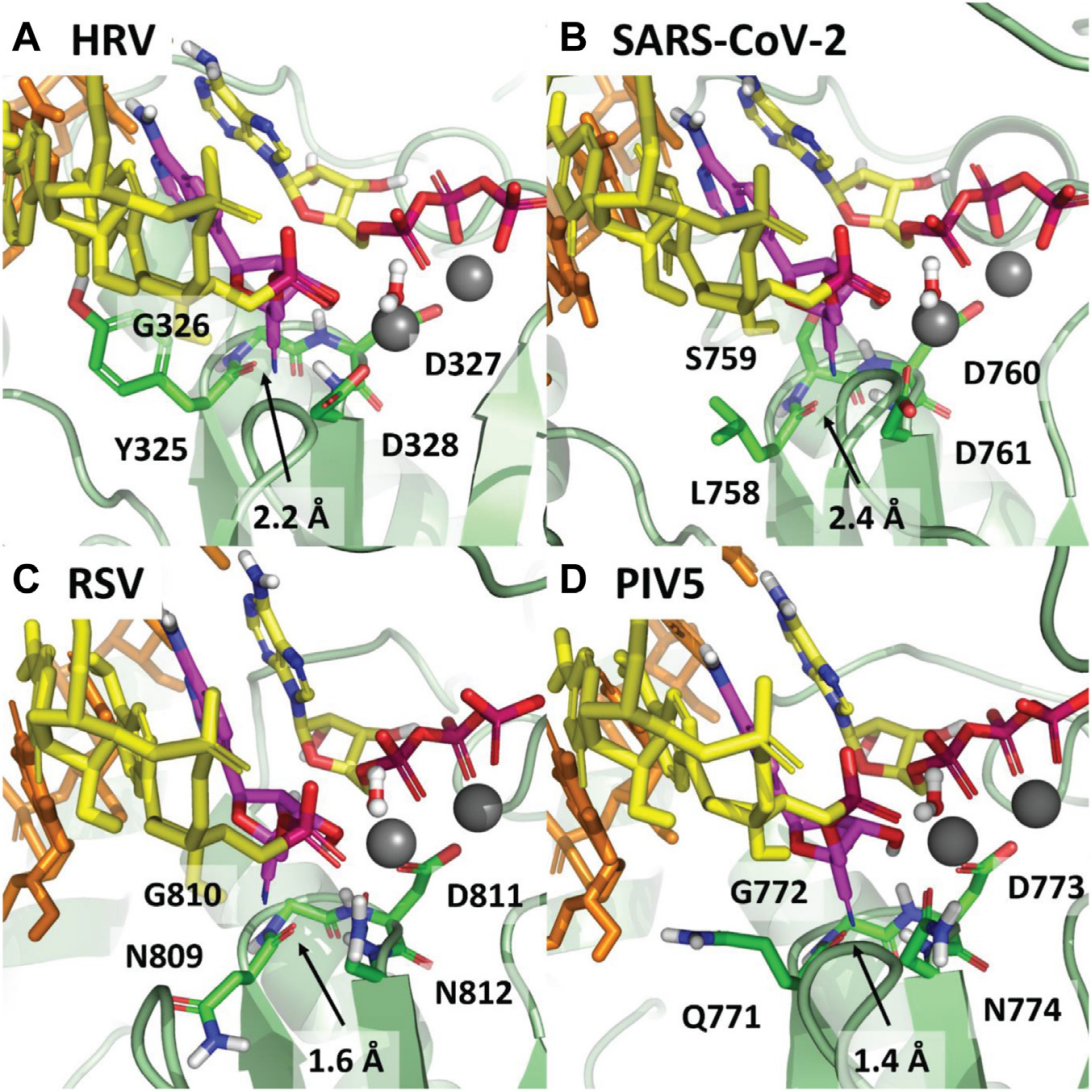
**Models of incorporatedGS-646939translocated to the “i + 1” position inHRV,SARS-CoV-2,RSV, and, PIV-5.** Structures are not optimized but serve as a guide to clashes that impair further incorporation, GS-646939 is shown in magenta. Following incorporation, translocation of the RNA to position the inhibitor in the first primer position is hindered by residues of motif C which coordinate the catalytic metals. For HRV (*A*), Cα of Gly-326 and NH of Asp-327 present a translocation obstacle to the 4ʹ-cyano of the incorporated inhibitor. A similar impediment to translocation exists for SARS-CoV-2 (*B*). Assuming translocation occurs, C=O of Tyr-325 presents a significant steric clash which would perturb proper primer positioning. This clash appears to be more significant in RSV (*C*) and PIV5 (*D*). HRV, human rhinovirus; PIV-5, parainfluenza virus 5; RdRp, RNA-dependent RNA polymerase; RSV, respiratory syncytial virus; SARS-CoV-2, severe acute respiratory syndrome coronavirus 2.

An analysis of the trajectory of the incorporated GS-646939 during translocation from the “i” substrate position to the “i + 1” primer position may help to provide a better understanding of the requirements for chain termination. For all the RdRps studied here, the 4ʹ-cyano moiety of the inhibitor must pass through a set of motif C residues, which coordinate the two catalytic metals (Fig. 7). The obstacle presented by these residues is largely independent of the specific side chains and instead derives from the protein backbone itself. For HRV (Fig. 7*A*) and SARS-CoV-2 (Fig. 7*B*), this clash is moderate. But for RSV (Fig. 7*C*) and PIV5 (Fig. 7*D*), the clash appears to be severe, significantly impairing proper positioning of the primer. If translocation is indeed compromised, one would expect diminished NTP incorporation independent of the nature of the incoming nucleotide. Like GS-646939, 4ʹ-ethynyl-2-fluoro-2ʹ-deoxyadenosine (EFdA) possesses a bulky 4ʹ-modification and was shown to block translocation of HIV-1 reverse transcriptase (HIV-1 RT) (50, 51, 52). Therefore, we used EFdA as a benchmark. For HRV-16 RdRp, GS-646939- and EFdA-terminated primers generally reduce NTP incorporation of all nucleotides tested (Fig. S7). High concentrations of UTP and 2-thio-UTP are required to obtain the “i + 1” product, while 3ʹdeoxy UTP, 2ʹO-methyl UTP, and 2′deoxy UTP are not incorporated. In contrast, AMP- and GS-443902-terminated primers do not cause immediate chain termination and instead enable the incorporation of each of these nucleotide analogs to a similar extent.

### Significance of template-dependent inhibition by GS-646939

**Figure 8:**
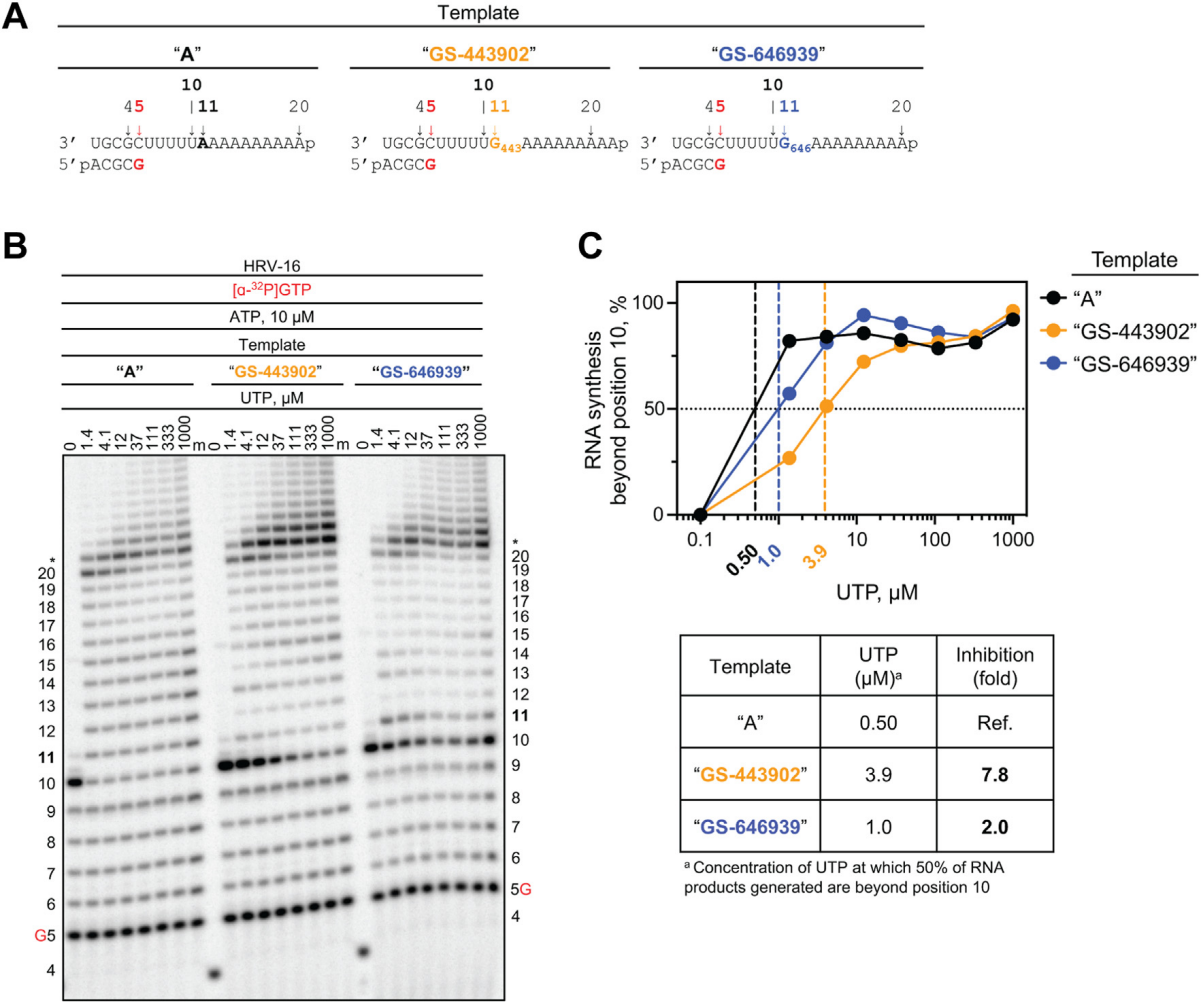
**RNA synthesis catalyzed by HRV-16 RdRp using a template with a single GS-443902 or GS-646939 residue embedded in the template at position 11**. *A*, RNA primer/template with an embedded GS-443902 (*Template “GS-443902”, middle*) or GS-646939 (*Template “GS-646939”, right*); the corresponding primer/template with adenosine (*Template A*) at this position is shown on the *left*. G5 indicates incorporation of [α-^32^P]-GTP at position 5. *B*, migration pattern of the products of RNA synthesis catalyzed by HRV-16 RdRp. MgCl_2_, [α-^32^P]-GTP, and ATP provided to the reaction to support RNA synthesis up to position 10. Increasing concentrations of UTP were supplemented to the reactions to monitor incorporation opposite a templated adenosine, GS-443902, or GS-646939 at position 11, and templated adenosines from position 12 to 20. Compared to Template A, intermediate products form at position 10 on Template “GS-443902” and “GS-646939”, indicating template-dependent inhibition. Product formation at and beyond the *asterisk* indicates RNA products that are likely a result of sequence-dependent slippage events. A 5′-^32^P-labeled 4-nt primer serves as a size marker. *C*, quantification of *B* (*top*) where the sum of RNA products generated beyond position 10 was divided by the total signal in the lane and normalized as a percentage, fold-inhibition resulting from an embedded GS-443902 or GS-646939 (*bottom*). To account for template-dependent differences in activity, product fraction was normalized as a percentage to product fraction observed at 1000 μM UTP for that template. HRV, human rhinovirus; RdRp, RNA-dependent RNA polymerase.

For SARS-CoV-2 RdRp, it has been demonstrated that higher NTP concentrations can overcome GS-443902-induced delayed chain termination or pausing (23, 26, 27, 31). This in turn may lead to full-length products containing embedded nucleotide analogs. When used as a template, UTP incorporation opposite the complementary GS-443902 is diminished. This unified template-dependent inhibition mechanism for GS-443902 has been described for several viral RdRps (27). Here, we designed RNA templates to monitor UTP incorporation opposite a template-embedded GS-443902 or GS-646939 residue, respectively (Fig. 8*A*). For the HRV-16 RdRp, submicromolar UTP concentration was sufficient to generate full-template length product on template “A” that contains the natural nucleotide (Fig. 8*B*). Comparatively, on templates “GS-443902” and “GS-646939”, inhibition opposite the embedded analog could be rescued by increasing UTP concentrations ∼8- and 2-fold, respectively (Fig. 8*C*). Similarly, EV-71 RdRp-catalyzed UTP incorporation was inhibited to a greater extent across GS-443902 (∼16-fold) than GS-646939 (∼9-fold) (Fig. S8). For SARS-CoV-2 and MERS-CoV RdRp, RNA synthesis opposite GS-646939 generates multiple intermediate products, indicative of an inhibitory effect (Fig. S9). To conclude, GS-443902 and GS-646939 cause inhibition in primer extension reactions when embedded in the template. While the template-dependent mechanism seems to be the dominant mode of inhibition of RdRp enzymes by GS-443902, GS-646939 acts primarily as a chain terminator.

## Discussion

The availability of effective antivirals for treating infections with respiratory RNA viruses is limited. Nucleotide analog RdRp inhibitors have the potential to act broadly against diverse RNA viruses, which is of particular importance in outbreak situations with emerging pathogens. Mutagenic nucleotides such as ribavirin, favipiravir, or molnupiravir provide prominent examples in this regard (53, 54, 55, 56, 57, 58). These compounds are base-modified, which can cause lethal mutagenesis when the active triphosphate metabolite is used as a substrate (46, 59). Classic nucleotide analogs with modifications in the sugar moiety commonly inhibit RNA synthesis. Here, we studied the mechanism of action of GS-646939, the nucleotide triphosphate metabolite of the newly discovered GS-7682 4ʹ-cyano modified *C*-adenosine nucleotide phosphoramidate analog. Inhibition of RNA synthesis was evaluated against an array of purified recombinant RdRp enzymes representing medically relevant respiratory RNA viruses. The broadly acting 1ʹ-cyano modified *C*-adenosine nucleotide triphosphate analog GS-443902 was included in this study for comparative purpose. Despite a certain degree of overlap in the spectrum of antiviral activities, the results of this study demonstrate distinct mechanisms of action for GS-443902 and GS-646939. While GS-443902 inhibits RNA synthesis predominantly when embedded in the template strand (27, 45), GS-646939 causes chain termination at the site of incorporation.

For RNA polymerases evaluated in this and our previous studies, the data reveal a decrease in selectivity for GS-443902 in the order of SARS-CoV-2, SARS-CoV, MERS-CoV > HRV-16, EV-71 > RSV, HMPV > HPIV-3, PIV-5 > LASV > influenza B ≫ h-mtRNAP. GS-443902 is a better substrate for the SARS-CoV-2 RdRp complex than its natural counterpart ATP (24, 26). Cryo-EM structures of the enzyme complex with bound RNA and a preincorporated GS-443902 revealed a hydrophilic pocket composed of Nsp12 Thr-687, Asn-691, and Ser-759 that accommodates the 1ʹ-cyano group (25). Of note, the S759A mutation was shown to confer resistance to GS-5734, due to a 5- to 10-fold increase in discrimination against GS-443902 (25, 48). Thus, biochemical and structural data align with *in vitro* selection experiments in explaining the selectivity for GS-443902 over ATP.

The preference for selective incorporation of GS-646939 follows the order: HRV-16 and EV-71 > SARS-CoV-2 and MERS-CoV ≈ RSV and HMPV > HPIV-3 and PIV-5 ≫ LASV ≫ influenza B = h-mtRNAP. For HRV-16 and EV-71 RdRp, GS-646939 is used ∼20 to 50 fold more efficiently as a substrate than ATP. To the best of our knowledge, this is the most favorable selectivity ever reported for the incorporation of a nucleotide analog by viral polymerases. Modeling of the preincorporated GS-646939 indicates that the 4ʹ-cyano is tolerated by each of the aforementioned polymerases with the exception of LASV, influenza B, and h-mtRNAP. With respect to HRV-16 and EV-71 RdRp, the particular combination of residues that form both the 2ʹ-OH recognition motif and the 4ʹ pocket allow for a favorable interaction between the 4ʹ-cyano and Asn-296 in HRV. In the case of LASV and influenza B, both the 1ʹ-cyano of GS-443902 and the 4ʹ-cyano of GS-646939 disrupt a water-mediated hydrogen bonding network which serves to recognize the ribose 2ʹ-OH. The active site of h-mtRNAP is dissimilar to any of the viral RdRps, with predicted clashes for both 1ʹ-cyano and 4ʹ-cyano substitutions.

GS-443902-mediated inhibition of primer extension reactions is generally heterogeneous (45). The position and extent of inhibition depend upon the nature of the polymerase, and the underlying structural determinants have not yet been investigated for RSV and other RdRps. For the SARS-CoV-2 RdRp complex, delayed chain termination and backtracking have been associated with the incorporation of GS-443902 (26, 31). Although structural evidence for backtracking has been reported (60), it is currently not known whether these events can be linked to each other and whether GS-443902 triggers similar effects also on other viral polymerases. In contrast, all viral RNA polymerases tested toward this end inhibit the incorporation of UTP opposite an embedded GS-443902 in the template. This inhibition may not necessarily translate into significant antiviral effects because the incorporation of GS-443902 may be inefficient in some cases, as it is for LASV and influenza viruses (45). For GS-646939, a unifying mechanism of action is chain termination. The extent of inhibition depends on the NTP concentration that can overcome blockages. Chain termination with HRV-16 and EV-71 RdRp is indeed not absolute, and higher NTP concentrations allow the continuation of RNA synthesis. However, the high selectivity for GS-646939 over ATP seems sufficient to cause potent antiviral effects. Chain termination with SARS-CoV-2 and MERS-CoV is also overcome with higher NTP concentrations, but a limited antiviral effect in cellular assays may be explained by the presence of a viral exonuclease, which can remove the incorporated inhibitor and allow synthesis to continue (61). For RSV and HMPV, chain termination is nearly absolute and lower nucleotide analog incorporation rates are therefore still sufficient for potent antiviral effects.

The antiviral effects and biochemical properties of several other nucleotide analogs with bulky 4ʹ-modifications have been reported, and chain termination is the dominant mechanism of action (52, 62, 63, 64, 65, 66, 67, 68, 69). Prodrugs of 4ʹ-azidocytidine (balapiravir) and 4ʹ-chloromethyl-2ʹ-deoxy-2ʹ-fluoro-cytidine (Lumicitabine) were developed for the treatment of hepatitis C virus and RSV infection, respectively. EFdA was developed for the treatment of HIV-1 infection. EFdA-TP is efficiently incorporated by HIV-1 RT, and structural data provide strong evidence to show that chain termination is based on compromised enzyme translocation (50, 51, 52). A hydrophobic pocket accommodates the 4ʹ-ethynyl moiety, which facilitates binding of EFdA-TP and stabilizes the complex after incorporating the nucleotide analog. The favorable interactions in the pre-translocated state and unfavorable interactions in the post-translocated state essentially block RT translocation and further incorporation events. Our structural models of RdRp enzymes considered in this study point to a similar mechanism with GS-646939. In contrast, 4′-fluorouridine was shown to cause delayed chain termination against RSV RdRp (62). These data suggest that the relatively small 4′-fluoro modification does not interfere with the translocation at the point of incorporation.

In conclusion, 1ʹ-cyano and 4ʹ-cyano modified nucleotide analogs inhibit diverse RdRp enzymes of respiratory RNA viruses *via* different mechanisms. Enzymes of the *Coronaviridae* (SARS-CoV-2 and MERS-CoV) and the *Pneumoviridae* (RSV and HMPV), are effectively targeted by the 1ʹ-cyano modified *C*-adenosine GS-443902. Selective incorporation, various blockages during primer extension, and efficient template-dependent inhibition provide biochemical explanations for the observed antiviral activity (23, 26, 27, 31). RdRps of the *Picornaviridae* (HRV-16 and EV-71) and *Pneumoviridae* (RSV and HMPV) are effectively targeted by the 4ʹ-cyano modified *C*-adenosine GS-646939. Selective incorporation and efficient chain termination were identified here as important parameters that correlate with potent antiviral activity observed in cell culture (32). The described inhibitory effects were observed with two prototypic polymerases of the *Coronaviridae*, the *Pneumoviridae* and the *Picornaviridae*; hence, the data provide confidence that the 1ʹ-cyano and 4ʹ-cyano modified nucleotides can be considered broadly to target emerging viruses that belong to these families.

## Experimental procedures

### Nucleic acids and chemicals

RNA primers and templates used in this study were 5′-phosphorylated and purchased from Dharmacon. GS-646939 and GS-443902 were provided by Gilead Sciences. NTPs were purchased from GE HealthCare. [α-^32^P]GTP was purchased from PerkinElmer (Revvity). 3′deoxy UTP, 2′O-methyl UTP, and 2-thio UTP were purchased from TriLink Biotechnologies. 2′deoxy UTP was purchased from MedChem Express.

### Expression and purification of viral polymerases

Expression and purification of MERS-CoV, SARS-CoV-2, RSV, LASV, FluB, and h-mtRNAP used in this study have been described (23, 26, 27). The baculovirus expression system was used for HMPV, HPIV-3, and PIV-5 RdRp P/L complex. The pFastBac-1 (Invitrogen) plasmid with the codon-optimized synthetic DNA sequences (GenScript) coding for HMPV (L: AAQ67700 and P: AAQ67693), were used as starting material for protein expression in insect cells (Sf9, Invitrogen). HMPV P/L complex was expressed as a polyprotein in frame with the N-terminal tobacco etch virus protease using the MultiBac (Geneva Biotech) (70, 71). This approach was originally reported for the expression of influenza virus RdRp trimeric complex (72, 73). P and L proteins were cleaved post translationally from the polyprotein at the engineered tobacco etch virus sites. HPIV-3 (L: NP_067153.2 P: 067149.1), and PIV-5 (L: AFE48451.1 and P: AFE48445.1) were cloned into the pFastBac Dual vector (Invitrogen), P was under the control of the p10 promoter and L under the polyhedrin promotor. The RdRp P/L complexes were purified using Ni-NTA affinity chromatography based on their respective eight-histidine tag as shown in Table 1 (Thermo Fisher Scientific). The pET-15b (Novagen) plasmid with codon-optimized synthetic DNA sequences (GenScript) coding for the RdRp of EV-71 (BAJ49823.1) and HRV-16 (AAA69862.1) were expressed in *Escherichia coli* BL21. EV-71 and HRV-16 RdRp enzymes were purified using Ni-NTA affinity chromatography based on their respective C-terminal eight-histidine tag according to the manufacturer’s specifications (Thermo Fisher Scientific). The protein identities of the purified HMPV, HPIV-3, and PIV-5 RdRp P/L complex as well as EV-71 and HRV-16 RdRp were confirmed by mass spectrometry analysis (The Alberta Proteomics and Mass Spectrometry Facility, University of Alberta, Canada).

### Evaluation of GS-646939 and GS-443902 incorporation and subsequent primer- and template-strand inhibition on viral RNA synthesis

The following synthetic 5′-monophosphorylated RNA templates were used in this study (the portion of the template that is complimentary to the 4-nt primer is underlined): 3′TGCGCTAGTTT for h-mtRNAP selectivity values; 3′UGCGCUAGAAAAAAp for MERS-CoV, SARS-CoV-2, EV-71, HRV-16, FluB, LASV analog selectivity, ATP/GS-646939 and GS-646939/GS-443902 competition, pattern of inhibition, and UTP and UTP analog incorporation at position “i + 1” experiments; 3′UGCGCUAGUUUAUUp for RSV, HMPV, HPIV-3, and PIV-5 analog selectivity, pattern of inhibition, and UTP incorporation at position “i + 1” experiments; 3′UGCGCUUUUUAAAAAAAAAAp, 3′UGCGCUUUUUG_646_AAAAAAAAAp, and 3′UGCGCUUUUUG_443_AAAAAAAAAp were used for the evaluation of nucleotide incorporation opposite GS-646939 (Template “GS-646939”) and GS-443902 (Template “GS-443902”). RNA synthesis assays of viral RdRp, data acquisition, and quantification were performed as previously reported by us (23, 26, 27, 45, 46, 48, 74). Briefly, viral RdRp concentration was optimized to ensure incorporation of [α-^32^P]-GTP is within the linear range. For the evaluation of single nucleotide incorporation, the concentration range of ATP, GS-646939, and GS-443902 were optimized to avoid misincorporation at subsequent positions. Reaction mixture for RNA synthesis assay (final concentrations after mixing), contain the purified viral RdRp, Tris–HCl (pH 8, 25 mM), RNA primer (200 μM), RNA template (2 μM, except for FluB RdRp reactions where optimal RNA template concentration was 0.5 μM), [α-^32^P]-GTP (0.1 μM), and various nucleotide concentrations were prepared on ice and incubated at 30 °C for 5 to 10 min. RNA synthesis was initiated by the addition of 5 mM MgCl_2_, except for SARS-CoV-2 and MERS-CoV RdRp, which required 1.25 mM MgCl_2_. The duration of reactions varied among the enzymes, SARS-CoV-2, MERS-CoV, HRV-16, EV-71, and h-mtRNAP reactions were stopped after 10 min; for RSV, HMPV, HPIV-3, PIV-5, FluB, and LASV reactions were stopped after 30 min. All reactions were stopped by the addition of equal volume formamide/EDTA (50 mM). The reaction products were incubated at 95 °C for 5 min and then resolved by 20% Urea-PAGE, and the [α-^32^P] generated signal was stored and scanned using Typhoon phosphorimager (Cytiva). The data were analyzed using GraphPad Prism 7.0 (GraphPad Software, Inc, https//www.graphpad.com).

### Generation of structural models

Models of nucleotide incorporation for representative enzymes in this study were generated based on existing X-ray or cryo-EM structures. For SARS-CoV-2, models were based on the cryo-EM structure 7UO4 (25), which includes a template, a 3′deoxy-primer, preincorporated GS-443902, and a single Mn^++^ ion. A 3′OH was added to the primer, the Mn^++^ was changed to Mg^++^ and a second metal was added. The active site was then refined with MacroModel (Schrödinger) (75, 76) for incoming ATP, GS-646939, and GS-443902. The HRV-A16 model was based on the X-ray structure 4K50 (36), which includes only primer and template. Based on the SARS-CoV-2 model and an X-ray structure for the similar hepatitis C virus polymerase (4WTD) (77), the two metals were added, and the structure was refined for the same three NTP’s as above. The Lassa model was based on the cryo-EM structure 7OJN (43), which includes template, primer, two Mn^++^ ions, and a nonhydrolyzable UTP analog. In this case, some ambiguity in how the NTP 2′OH was recognized by the enzyme suggested to us that a bridging water may be involved. To answer this question, a WaterMap (Schrödinger) (78, 79) analysis was run, which indicated that a single water molecule linked the NTP 2′OH to the active site residues His-1298 and Ser-1330. A similar situation arose for FluB, which was based on the X-ray structures 6QCV and 6QCX (42). In this case, we found a single water molecule linked the NTP 2′OH to the active site residues Asn-414 and Ser-443. The model for h-mtRNAP was based on the X-ray structure 4BOC (44), which includes primer and template. Again, we took a similar approach to optimizing for incoming ATP, GS-443902, and GS-646939 as above. More challenging were the models for RSV (based on the apo cryo-EM structure 6PZK (38) and PIV-5 (based on the apo cryo-EM structure 6V85 (40)). These structures required major modifications to accommodate the primer, template, metals, and NTP. Similarity in the structure to Lassa allowed us to insert those elements from that model into the apo structure for RSV. The protein was then optimized around the RNA, using side chain sampling and minimization in Prime (Schrödinger) (80, 81), before allowing the RNA to also relax using Macromodel. Side chain conformations in the active site were modified following the precedent of the above structures to recognize the metals and the NTP. Notably, active site motif A (residues 700–705) underwent a significant conformational change from its apo form. Investigation of multiple side chain conformations to recognize the NTP 2′OH ultimately settled on a configuration in which Met-705 and Gln-782 directly hydrogen bond to the ribose. No bridging water molecule was predicted. The optimization of the PIV5 model followed a similar path.

## Data availability

All data are included within this article.

## Supporting information

This article contains Supporting information (45, 47).

## Conflict of interest

M. G. received funding from 10.13039/100005564Gilead Sciences in support for studies on the mechanism of action of nucleotide analog polymerase inhibitors. J. P., D. S. S., J. K. P., J. Y. F., and J. P. B. are current or former Gilead employees.
